# Machine Learning Models for Objective Assessment of Vascular Anastomoses Using Computational Fluid Dynamics for Surgical Skill Training—A Retrospective Study

**DOI:** 10.3390/jcm15103588

**Published:** 2026-05-07

**Authors:** Levente Kiss-Pápai, Stefánia Reich, Júlia Varga, Wouter Oosterlinck, Peter Gloviczki, Balázs Gasz

**Affiliations:** 1 Institute of Transdisciplinary Discoveries, University of Pécs, Szigeti út 12., 7624 Pécs, Hungary; 2 ME3D-Graft Ltd., Őz utca 5., 7624 Pécs, Hungary; 3 Department of Cardiovascular Sciences, KU Leuven, Herestraat 49 box 911, 3000 Leuven, Belgium; 4 Division of Vascular and Endovascular Surgery, Mayo Clinic, Rochester, MN 55905, USA

**Keywords:** skill training, machine learning, surgical education, vascular surgery, performance assessment

## Abstract

**Background:** Objective performance assessment is essential in surgical skill training, yet current methods are labor-intensive and focus on observing the trainee rather than the end-product of the procedure. Machine learning (ML) methods offer reproducible feedback but have mainly relied on kinematic or video data, often reducing assessment to binary or ternary classification. Our objective was to compare ML regression models predicting expert-assigned scores of vascular anastomoses from computational fluid dynamics (CFD) features of the final product. Additionally, we aimed to assess biomechanical plausibility of predictions. **Methods:** A total of 146 participants performed 419 end-to-side anastomoses on case-specific three-dimensional (3D) printed simulators. Anastomoses were digitized via 3D scanning, ranked by experts, and characterized using CFD-derived hemodynamic features. These served as input for linear models (Ridge, Partial Least Squares), support vector machines, and tree-based ensembles (Random Forest, Extremely Randomized Trees, and Extreme Gradient Boosting [XGBoost]), evaluated using 10-fold nested cross-validation with genetic hyperparameter optimization. **Results:** Inter-rater reliability of expert indicated strong agreement (intraclass correlation coefficient ICC3k = 0.846). XGBoost achieved the lowest mean root mean squared error of 0.758 (95% bootstrap CI: 0.722–0.799) and a coefficient of determination (R^2^) of 0.673 (0.617–0.725), with the most stable performance across folds. Shapley additive explanations (SHAP) identified the wall shear stress gradient, transverse wall shear stress, and maximum pressure as the most influential features—variables associated with intimal hyperplasia and atherosclerotic remodeling. **Conclusions:** Tree-based ensemble methods, particularly XGBoost, effectively modeled biomechanical properties against expert scores. Combining CFD and ML can provide reproducible, mechanistically relevant feedback in vascular surgical skill training.

## 1. Introduction

Skill training has become an essential part of the surgical curriculum and continuing education since a shift towards competency-based education with an increased awareness of patient safety and quality control [1,2]. Participant assessment after training sessions is an important aspect of training programs, allowing both formative and summative feedback. While many cardiovascular and vascular skill training products have appeared lately, most of them lack an independent assessment capability [3]. Instead, trainees tend to be individually supervised by expert tutors in order to provide a manual assessment of the given training session, posing an important resource problem.

To reduce subjectivity in manual assessment, multiple Likert-based global rating scales (GRSs) have been developed. In most study settings, these generally conclude the Objective Structured Assessment of Technical Skills (OSATS) score [4,5,6] or subdiscipline-specific adaptations of it [7,8,9,10,11]. Such assessment tools rate general dexterity metrics, overall confidence, procedure time, and, in some versions, suturing technique (e.g., completeness of dissection, bleeding, tissue planes, and suture tension). While these methods have been claimed to be valid tools in assessing the trainee, the assessment process itself is labor-intensive and requires a trained evaluator [4,12].

To deal with the drawbacks of manual scoring, such rating scales have been recently modeled and approximated by machine learning (ML) algorithms. In prior ML models, mainly kinematic sensor data or video data were chosen as input, to match the distinct questions of a GRS scale. An important drawback of many of these studies is that they only solve a binary or ternary classification problem, grouping trainees into 2–3 expertise levels only (beginner, medium, and expert), and thus fail to give granular feedback [13,14,15,16]. Other studies are built on regression models for predicting the OSATS score directly; however, they are based on a limited sample size [17]. ML models that utilize information from the final product (such as an anastomosis) are completely lacking in education settings, even though some studies show that it would give a close correlation with the OSATS or other GRS scores [9,18]. Minimally invasive, robotic skill assessment uses validated tools such as Robotic-OSATS, GEARS [19], providing expert-based evaluation of technical performance during standardized robotic training tasks, while the da Vinci Skills Simulator generates automated metrics including time, economy of motion, collisions, excessive force, and missed targets [20].

Moreover, evaluating the end-product could form a link between simulation-based performance and real-world proficiency, as several biomechanical properties have been linked to pathophysiology in vascular structures. Computational methods have already been extensively used in aneurysm pathogenesis and prognosis modeling and have been applied in the clinical practice for risk prediction [21,22]. Similarly, fluid dynamics properties have been associated with important pathological anastomosis remodeling, histological and medical outcomes, such as intimal hyperplasia, plaque formation, or re-stenosis [23,24,25,26,27,28,29,30,31]. Biomechanics has also been put to use for trainee assessment through directly measuring in vitro energy loss and pressure loss in wet-lab situations [32].

We hereby propose a model that applies the method of computational fluid dynamics and ML to give an objective and explainable assessment for cardiac and vascular surgical simulation results based on the final product (anastomosis) of a training session.

## 2. Materials and Methods

### 2.1. Skill Training

The training sessions were conducted on various 3D-printed case-specific simulators, with the objective to perform an arterial anastomosis in an end-to-side (EtS) configuration. Participant experience level was not an exclusion criterion. Experience levels were included based on voluntary consent.

### 2.2. Digital Geometry Reconstruction, Numerical Simulation Conditions

The anastomoses were digitized according to Wlasitsch et al. [33]. Briefly, the inner volume (lumen) was reconstructed and 3D-scanned in a resolution of 10 µm (Cara 3.2 scanner, Kulzer-Dental Inc., Tokyo, Japan). The scanned specimens were then optimized and volume mesh was generated for CFD analysis.

The CFD simulations were carried out as described by Wlasitsch et al. on the basis of blood flow modeling in 3D; governing equations were continuity and Navier–Stokes equations (see in details in Supplementary Information S1) [33].

Wall shear stress (WSS) is defined as the tangential force exerted by blood on the vessel wall per unit area. Multiple WSS-based indicators have been calculated, i.e., the time-averaged wall shear stress (TAWSS) and gradient (WSSG), Oscillatory Shear Index (OSI), Relative Residence Time (RRT), and transverse WSS (WSStrans). OSI is intended to reflect the direction and intensity changes in blood flow, while RRT marks the residence time of blood on the arterial wall. WSStrans represents the component of wall shear stress acting perpendicularly to the predominant direction of blood flow. Energy loss and energy efficiency were calculated as described in the Supplementary Materials Equations (S8) and (S10). As the fluid is incompressible, pressure drop and energy loss are related linearly. Further variables were the velocity (v), flow rate (Q), pressure (p), and pressure drop between inlets and the outlet (dp). The operationalization of variables with temporal and/or spatial dimensions was through the median, if not indexed otherwise. For the exact formulas regarding the hemodynamic properties, please refer to the Supplementary Materials.

### 2.3. Pilot Study and Manual Labeling

In a preliminary experiment, 30 trainees were randomly selected from various experience levels and multiple institutes. 3D visualizations of end-to-side vascular sutures performed by trainees on skill training courses (see Section 6) were shown to domain experts, to rank their quality based on their judgment, while taking into consideration the overall structure, morphology and characteristic alterations (e.g., deep stitches, wall inversions, bifurcation angle, and bulging or incavations of the wall). Assessors were instructed to consider the severity and number of characteristic alterations, and ultimately to relate the overall structure to their clinical experience and previously ranked samples. After the pilot study, assessors discussed the pilot dataset (without altering ranks) to better align for future assessments. The relationship among the sample-wise average of ranks, clinically relevant fluid dynamics properties, and trainee years of experience were measured using Spearman’s rank correlation to assess the validity of the ranks. Inter-annotator reliability was quantified using the mean intraclass correlation coefficient applied for 3 raters (ICC3k), implemented in the scipy 1.10.0 and pingouin 0.5.3 Python packages. The pilot cases and their average ranks were made available to the annotators to help with further labeling after the pilot study.

Each anastomosis 3D mesh was evaluated by at least two annotators independently. The scores were averaged sample-wise and used as the single outcome variable for training the ML models. This has been the chosen method in order to decrease assessment uncertainty, and it is notable that this outcome variable reflects expert consensus rather than an external gold standard. The study design was reviewed by the University of Pécs, and ethical approval has been granted under the reference number 9718/PTE2023.

### 2.4. ML Model Selection and Training

Only CFD features were included in the models, i.e., the algorithms were blind to the used simulator, actual level of experience or other characteristic properties of the simulator and participant.

The model pool included regularized linear models (Ridge; Partial Least Squares, PLS), support vector machine regression (SVR; using linear, polynomial, or radial basis function kernels), and decision tree ensembles (Random Forests, RF; Extremely Randomized Trees, XT; Extreme Gradient Boosting, XGBoost). Regularized linear models help to control model complexity and account for multicollinearity in the dataset. Support vector machines is a kernel-based method that transforms the original variable space into a higher dimensional “feature space”, thus allowing for modeling non-linear relationship. Decision tree ensembles build on the idea of combining multiple “weak learner” decision trees, with RF and XT building trees independently from one another on bootstrapped samples, and with XGBoost building decision trees consecutively based on the errors of previous trees.

Each estimator had its distinct hyper-parameter search space (summarized in Supplementary Materials Table S1), and each estimator was tuned with genetic search with adaptive, exponentially increasing crossover and decaying mutation probabilities over max. 30 generations using an initial population size of 30. Supplementary Materials Equation (S11) describes the adaptive probability function. An early stopping callback was used with a requirement of stagnating fitness values in ten successive generations. The evolutionary strategy was $\left(\mu +\lambda \right)-ES$, where $\left(\mu +\lambda \right)$ defines the total population size composed of μ parents and λ offsprings. μ=λ individuals are selected to be parents in the upcoming generation, thus the overall population size after the first generation was 60 in each generation.

**Figure 1:**
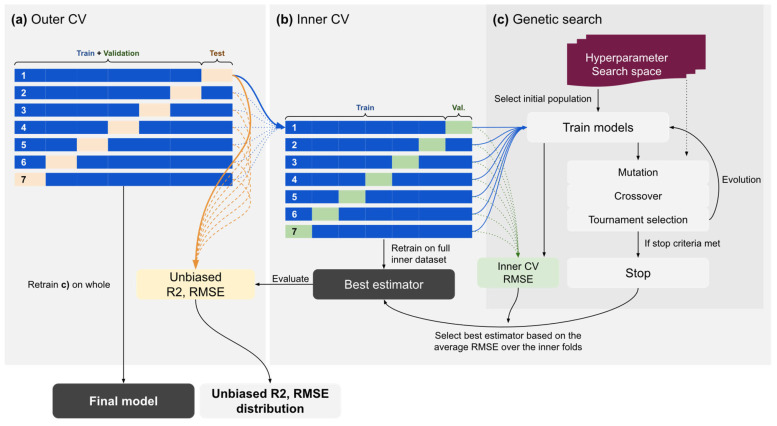
Schematic diagram of model development. Unbiased final performance estimate distribution was retrieved from the outer cross-validation (CV) loop (**a**). Hyperparameter search was conducted in the inner CV loop, and the estimator with the lowest mean squared error was returned for the outer loop to be evaluated (**b**). The hyperparameter space was explored using genetic search for max. 30 generations, with an early stopping mechanism (**c**).

Hyper-parameter tuning happened in the inner folds of a 10-fold nested cross-validation scheme, that is, an inner cross-validation loop for hyper-parameter tuning and model selection, and an outer loop of K-fold cross-validation for evaluating the model with unbiased estimates. Optimization was performed to minimize the mean squared error metric. The best-performing, hyper-parameter-tuned model was retrained and returned to be evaluated on the outer test fold. The method is summarized in Figure 1. As the evolutionary optimization independently selects hyperparameters in each cross-validation fold, pairwise statistical comparison between algorithms was not performed; instead, we report descriptive performance summaries across folds.

We implemented machine learning models and statistical calculations in Python 3.9, using Numpy 1.23.5, Scikit-learn 1.2.1, Sklearn-genetic-opt v.0.10.0, Scipy 1.10.0, Vtk 9.1.0, and Vmtk 1.5.0; visualizations were performed using Matplotlib 3.7.0 and Seaborn 0.12.2.

### 2.5. Evaluation

The data annotation was evaluated by measuring inter-rater consistency with a benchmark of ICC3k ≥ 0.8.

Overall generalization performance was evaluated using 10-fold (nested) cross-validation, with optimization for minimizing the root mean squared error (RMSE). Additional complementary metrics (Mean Absolute Error, MAE; Mean Absolute Percentage Error, MAPE; coefficient of determination, R2; Probability of error being under 0.5 points, P(E < 0.5)) were also computed and provided.

For a fuller understanding, cross-validated out-of-sample RMSE means were compared among the different model types, and the Shapley additive explanation (SHAP) method was employed to examine the importance of features and comprehend the models.

## 3. Results

### 3.1. Pilot Study

In the pilot study, a total of 83 anastomoses on the simulators (Table 1) were performed by 30 participants from multiple countries and institutes (Table 2). Several computational fluid dynamics variables showed moderate correlation with the years of experience (measured since graduation, |rs| ranging 0.37–0.61) and moderate-to-strong correlation with the average manual scores per participant (|rs| ranging 0.37–0.82). Furthermore, we found a moderate positive correlation between the suture labels and experience years (rs(28) = 0.37, *p* = 0.044). Correlation coefficients are summarized in Supplementary Materials Table S2. Inter-rater agreement was above 0.75, which can be interpreted as a good level of agreement according to [34] (ICC3k = 0.875; CI95: 0.81–0.92).

### 3.2. Descriptive and Explorative Data Analysis

We expanded the pilot population to 419 end-to-side anastomoses (described in Table 1) completed by 146 participants (described in Table 2). Supplementary Materials Table S3 presents the distribution of the operationalized metrics obtained from the CFD simulations, which served as algorithm inputs, while their correlation is illustrated in Supplementary Materials Figure S1. Inter-rater reliability on the full dataset was ICC3k = 0.846 (CI95: 0.81–0.88).

### 3.3. Model Performance

**Figure 2:**
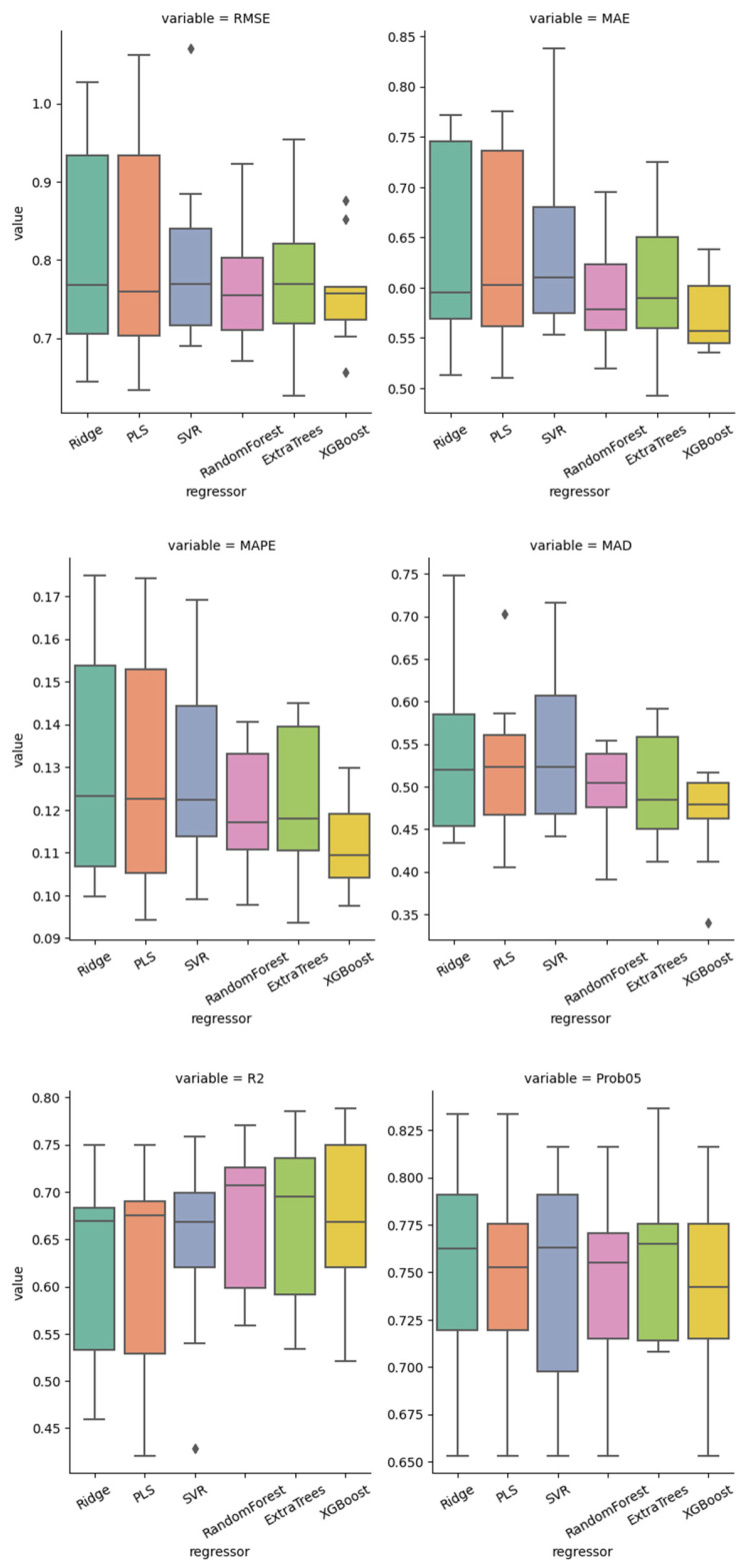
Performance comparison of the machine learning models based on the best estimator in the inner loop per model type. The diamonds mark outliers.

As described in Table 3 and Figure 2, non-linear algorithms outperformed the linear ones according to each (mean) metrics. More specifically, the XGBoost estimator achieved the lowest mean RMSE and the narrowest bootstrap confidence interval, indicating the most stable generalization performance, although RF and XT performed closely according to most metrics. Notably, the XT algorithm mildly outperformed in the probability of observing a low (<0.5 points) error.

**Figure 3:**
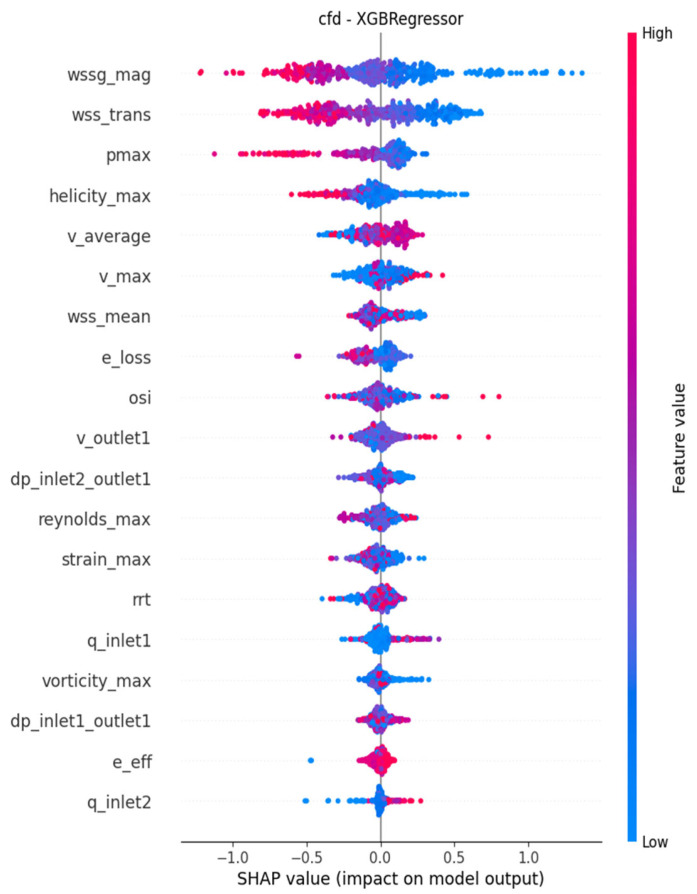
SHAP feature impacts in the XGBoost model.

**Figure 4:**
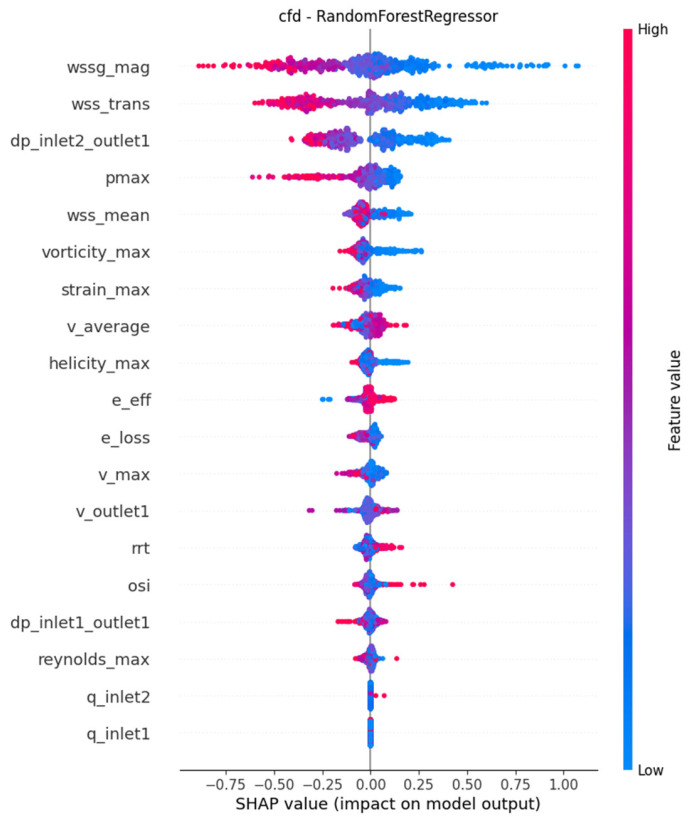
SHAP feature impacts in the Random Forest model.

**Figure 5:**
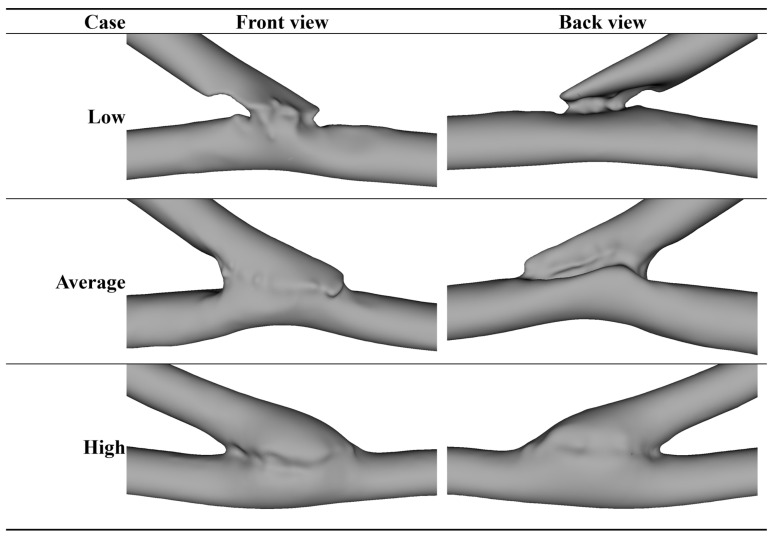
Example cases receiving the low (3.0), average (5.1), and high (9.5) scores.

As presented in Table 4, WSS_trans_, WSSG, dp_in2-out1_ and vorticity_max_ were consistently among the topmost important features across the models. These variables were approximately in a monotonous inverse relationship with the output variable based on Figure 3 and Figure 4, which illustrate the models with the first and second lowest RMSE metrics, respectively. As a visual illustration, three cases receiving low, average and high scores from the final model are represented in Figure 5.

## 4. Discussion

This study aimed to investigate a new method for reproducible surgical skill training assessment in vascular and cardiac surgical education based on a large sample of high-resolution 3D representations of actual hand-sewn end-to-side arterial anastomoses and their CFD simulations.

To confirm our fundamental hypothesis that marking the end result of a skill practice would correlate with indicators of experience, we conducted a pilot study with 30 trainees. The pilot concluded that the proposed manual labeling method is a valid and feasible way to discriminate surgical expertise levels, agreeing with prior studies [9,18]. Moreover, given that clinically important hemodynamic properties (such as different metrics derived from wall shear stress and energy loss) are also correlated with both the expert-given ranks and the years of experience, we argue that it is a suitable method for evaluating individual practice results in a meaningful way.

Following the successful pilot study, we expanded the scope and developed a machine learning pipeline to replace the manual labeling of structures. Our results show that tree-based ensemble methods were the most successful in predicting the manual scores, with XGBoost yielding the lowest mean error and the most consistent performance across folds. This may reflect the structure of the present dataset, namely its moderate sample size, correlated CFD features, and noisy continuous target, for which XGBoost’s sequential, residual-based training and explicit regularization are a reasonable fit, rather than a broadly generalizable advantage over RF or XT. The margins over RF and XT were nonetheless modest and their confidence intervals overlapped, so XGBoost is best understood as the most stable performer on this dataset rather than as categorically superior.

Based on SHAP values, WSS_trans_, WSSG and the pressure drop between the graft inlet and the outlet were consistently ranked as the most important input features determining the output of the models. Increased values of these parameters consequently led to worse anastomosis quality scores, which is in accordance with previous biomechanical research on maladaptive maturation. Specifically, high values of WSS_trans_ are associated with the development of intimal hyperplasia and atherosclerotic plaques [35,36,37,38,39,40], just like high WSSG values [35,41,42,43,44].

Performance assessment aimed at improving and objectifying surgical technical skills has become a major focus of modern surgical education. The widespread use of interconnected digital tools for data capture, transfer, and storage, described as the Internet of Surgical Things (IoST), has enabled more objective evaluation of surgical performance [45]. Previous studies have highlighted the role of telesurgery, telementoring, image-guided interventions, and sensor-based systems in measuring clinically relevant indicators such as path length, motion smoothness, fixation patterns, saccades, and procedural time [45,46]. Advanced algorithms have supported evaluation of efficiency, tremor control, depth perception, and economy of movement, with the ability to distinguish expertise levels [47]. However, these approaches often rely on fragmented sensing systems that provide only indirect representations of procedural quality [48]. Standardization, interoperability, and clinically outcome-oriented assessment remain limited, and more integrated, hybrid, and predictive models are needed to connect technical performance data with meaningful educational and clinical endpoints.

Compared to other automated techniques for evaluating surgical performance that rely on kinematic and video-stream data, this novel approach offers an important benefit by directly assessing the ultimate outcome of the surgical intervention using meaningful metrics that have been extensively researched and can be associated with the pathomechanisms of relevant diseases and adverse outcomes.

As surgical robotics continues to advance and partially autonomous instruments move closer to everyday clinical practice, complementary validation methods may become increasingly valuable for characterizing an instrument’s capabilities [49,50]. Beyond trainee assessment, our approach could potentially offer an outcome-based perspective that might help benchmark surgical equipment and inform the evaluation of such systems, although dedicated studies would be needed to confirm its applicability in this context.

Three-dimensional printing is becoming an increasingly important component of surgical planning and education, particularly in complex and patient-specific cases. Evidence from systematic reviews shows that 3D-printed models improve anatomical understanding, shorten decision-making time, and enhance training outcomes compared with conventional teaching methods [51]. Their applications extend from preoperative planning and intraoperative guidance to simulation, patient education, implants, and tissue engineering [52]. In training, realistic 3D-printed models have shown clear advantages over basic practice tools, with high trainee satisfaction regarding curriculum value and model fidelity [53]. In our work, 3D printing was specifically applied to create reproducible vascular simulation models that reflect individual anatomy, surgical topography, and site-specific technical challenges. Different but standardized scenarios were incorporated to reproduce surgical variability while preserving comparability between cases, thereby supporting objective measurement of anastomotic quality, reproducibility, and technical performance.

While CFD simulations are commonly used in engineering, they can become considerably more complex when dealing with elastic materials (such as arteries), non-Newtonian fluids (such as blood), or particles (such as blood cells or endothelial cells), leading to exponentially longer computation times. To reduce computational time and allow for timely feedback after training sessions, we made some reasonable simplifications in the numerical simulation conditions by assuming a rigid vessel wall and neglecting the simulation of individual particle components [54].

The rigid vessel wall assumption introduces systematic bias in absolute hemodynamic values. However, this assumption is applied uniformly across all samples, preserving the relative discriminative signal required by the machine learning models. In rigid-wall simulations, WSS values may be overestimated by 10–13% compared to compliant-wall models [55]; however, this does not skew the spatial distribution of shear stress [25], and in small-diameter or stiff vascular models, time-averaged rigid-wall results remain comparable to FSI results [54].

Besides CFD properties, geometrical properties are also linked to both biomechanical variables and pathophysiology of diseases [56,57,58]. Our future objective is to extract geometrical features from the 3D structures and explore their predictive ability and association with overall anastomosis quality in educational settings. Furthermore, geometrical analysis has already been effectively applied to predict fluid dynamics properties of vessels [59]. Therefore, geometrical feature vectors could also be used as input to, e.g., graph convolutional networks to estimate bio-mechanical attributes near the vessel wall for the trainees’ benefit. One major advantage of this approach would be that geometrical analysis does not require transient calculations; thus, computation time could be further reduced to provide swifter feedback for course participants.

## 5. Limitations

Although the results contribute to our understanding of vascular surgical performance, several limitations should be borne in mind when interpreting them. The CFD simulations assumed rigid vessel walls, constant outflow pressure, and omitted particle-level components. These simplifications were adopted to shorten computation time and enable timely feedback after training sessions, and their implications on absolute hemodynamic values have been addressed above. 3D meshes were uniformly scaled to the same inner diameters (3 mm), to improve generalizability on the cost of anatomical correctness. Only CFD-derived hemodynamic features were used as model inputs; geometrical descriptors of the 3D structures were not included, although, as outlined earlier, they represent a promising direction for future work and could further accelerate feedback by avoiding transient calculations. Because the evolutionary hyperparameter optimization independently selected configurations in each cross-validation fold, pairwise statistical comparisons between algorithms were not performed, and model ranking should therefore be interpreted in terms of mean out-of-sample error and the stability of its bootstrap confidence interval. The anastomoses were carried out on silicone simulators rather than biological tissue and only in the end-to-side configuration. Other anastomosis configurations differ structurally and fall outside the scope of the present model. Generalizability to in vivo conditions also remains to be established. Finally, despite good inter-rater reliability, the ground-truth scores ultimately depend on expert judgment. Although expert judgment was only influenced by the visualizations of the actual 3D structures, we acknowledge that the study was conducted with industry involvement, and thus external validation on independent datasets could be valuable to confirm the robustness and generalizability of the proposed models.

## 6. Conclusions

Surgical skill training must involve giving objective feedback after training sessions to be able to track progress and provide context for the individual. Testing several models, our study found that the Extreme Gradient Boosting algorithm can model the relationship between biomechanical properties and performance assessment. Combining CFD and ML techniques can provide reproducible, mechanistically relevant feedback in vascular surgical skill training and opens new perspectives for technical and non-technical education and training of minimally invasive and robot-assisted cardiovascular surgery. In this way, evaluator bias can be reduced, and reproducible feedback can be given with visual explanations, while also logging skill progress and providing the ability to re-evaluate new sessions using the same criteria.

## Figures and Tables

**Table 1:** Characteristic data of training cases. Counts represent number of cases.

	Pilot		Full	
**Suture type**				
6-0 polypropylene	80	96%	394	95%
7-0 polypropylene	3	4%	25	6%
**Diameter**				
3.0 mm	73	88%	375	90%
5.0 mm ^a^	10	12%	44	11%
**Simulator**				
Arteriovenous fistula operation ^b^	65	78%	297	71%
Eversion carotid endarterectomy on highly bifurcated left carotid artery	10	12%	45	11%
Coronary artery bypass graft (CABG ^c^) on LAD ^d^	8	10%	51	12%
N/A ^e^	0	0%	26	6%

^a^ d = 5.0 mm structures were scaled down to 3 mm during postprocessing, before numerical simulations. ^b^ instead of vein imitations, arteries were used in arteriovenous fistula surgery simulations in order to maintain a more homogeneous population for the CFD simulations. ^c^ CABG: coronary artery bypass graft. ^d^ LAD: left anterior descending artery. ^e^ lost information during processing.

**Table 2:** Demographic data of participants. Counts represent number of participants.

	Pilot		Full			Pilot		Full	
**Country**					**Sex**				
Hungary	19	63%	81	55%	Male	19	63%	93	64%
Serbia	3	10%	21	14%	Female	11	37%	52	36%
Switzerland	4	0%	15	10%	NA	0	0%	1	1%
Germany	3	10%	8	5%	**Specialization**				
Lithuania	1	3%	7	5%	Vascular surgery	12	40%	58	40%
Latvia	0	0%	4	3%	Cardiac surgery	2	7%	23	16%
Azerbaijan	0	0%	3	2%	General surgery	6	20%	20	14%
Bosnia and Herzegovina	0	0%	1	1%	Other	6	20%	20	14%
Poland	0	0%	1	1%	Orthopedic/trauma	3	10%	11	8%
USA–Illinois	0	0%	1	1%	Thoracic surgery	0	0%	4	3%
NA	0	0%	4	3%	NA	1	3%	10	7%
**Years of experience**					**Position**				
0–5	12	40%	61	42%	Resident	16	53%	90	62%
5–10	9	30%	15	10%	Consultant	13	43%	42	29%
10–15	4	13%	4	3%	NA	1	3%	14	10%
15–20	4	13%	4	3%					
20	1	3%	3	2%					
NA	0	0%	44	30%					

**Table 3:** Performance comparison of the machine learning models. Bold numbers mark the best performing model per the given performance metric.

	RMSE Mean (SE) (CI 95%)	MAE Mean (SE) (CI 95%)	MAPE Mean (SE) (CI 95%)	R2 Mean (SE) (CI 95%)	P(E < 0.5) Mean (SE) (CI 95%)
**Ridge**	0.818 (0.043) (0.737–0.909)	0.642 (0.030) (0.584–0.702)	0.130 (0.009) (0.114–0.147)	0.627 (0.034) (0.556–0.690)	0.748 (0.018) (0.711–0.783)
**PLS**	0.819 (0.046) (0.732–0.917)	0.640 (0.031) (0.579–0.703)	0.129 (0.009) (0.113–0.145)	0.626 (0.037) (0.546–0.692)	0.746 (0.017) (0.713–0.780)
**SVR**	0.800 (0.035) (0.739–0.879)	0.638 (0.027) (0.591–0.697)	0.128 (0.007) (0.115–0.141)	0.643 (0.030) (0.578–0.698)	0.744 (0.017) (0.710–0.776)
**RF**	0.769 (0.025) (0.722–0.820)	0.597 (0.018) (0.564–0.634)	0.119 (0.004) (0.111–0.128)	0.669 (0.024) (0.620–0.715)	0.746 (0.015) (0.716–0.773)
**XT**	0.771 (0.030) (0.713–0.832)	0.604 (0.024) (0.556–0.650)	0.121 (0.006) (0.110–0.132)	0.666 (0.028) (0.609–0.718)	**0.756** (0.014) (0.730–0.784)
**XGB**	**0.758** (0.021) (0.722–0.799)	**0.573** (0.012) (0.552–0.596)	**0.112** (0.003) (0.106–0.118)	**0.673** (0.028) (0.617–0.725)	0.742 (0.016) (0.711–0.772)

**Table 4:** Feature importance—SHAP values. Top 6 features per regression algorithm are displayed in bold.

	XT	RF	XGBoost	SVR	PLS	Ridge
**WSS_trans_**	**0.240**	**0.235**	**0.316**	**0.281**	**0.353**	**0.331**
**WSSG**	**0.205**	**0.253**	**0.327**	**0.430**	**0.379**	**0.357**
**dp_in2_out_**	**0.114**	**0.172**	0.066	**0.219**	**0.170**	**0.181**
**Vorticity_max_**	**0.089**	**0.059**	0.042	**0.122**	**0.075**	**0.128**
**TAWSS**	**0.086**	**0.062**	0.090	0.068	0.063	0.003
**Strain_max_**	**0.063**	0.043	0.065	0.071	0.011	0.039
**RRT**	0.050	0.028	0.064	**0.200**	**0.112**	**0.127**
**v_max_**	0.050	0.030	**0.092**	0.008	0.021	0.009
**Helicity_max_**	0.039	0.031	**0.137**	**0.232**	**0.116**	**0.115**
**v_average_**	0.031	0.041	**0.114**	0.026	0.012	0.014
**E_loss_**	0.031	0.030	0.084	0.019	0.053	0.021
**dp_in1-out_**	0.027	0.022	0.038	0.027	0.043	0.042
**p_max_**	0.023	**0.094**	**0.173**	0.052	0.011	0.026
**OSI**	0.018	0.023	0.082	0.040	0.053	0.048
**E_eff_**	0.016	0.031	0.033	0.107	0.015	0.061
**v_out_**	0.016	0.029	0.068	0.027	0.028	0.029
**Reynolds_max_**	0.011	0.014	0.065	0.007	0.041	0.033
**Q_in1_**	0.009	0.000	0.051	0.006	0.012	0.017
**Q_in2_**	0.007	0.000	0.032	0.030	0.059	0.047

## Data Availability

The data presented in this study are not publicly available due to proprietary restrictions related to ME3D-Graft Ltd. Reasonable requests for access may be considered by the corresponding author, subject to approval by the company.

## Supplementary Materials

The following supporting information can be downloaded at https://www.mdpi.com/article/10.3390/jcm15103588/s1, Supplementary Information S1: Detailed description of the methodology of skill training and numerical simulations; Table S1: Hyperparameter search space; Table S2: Spearman’s correlations among different CFD and trainee characteristics; Table S3: Descriptive statistics of numeric variables on the full dataset; Equations; Figure S1: Spearman’s correlation heatmap of the model variables.
