# Supplementary material for: Machine Learning Models for Objective Assessment of Vascular Anastomoses Using Computational Fluid Dynamics for Surgical Skill Training—A Retrospective Study
Source: J Clin Med. 2026 May 7;15(10):3588. doi: 10.3390/jcm15103588 (PMC13207516; doi:10.3390/jcm15103588)
Supplement: Supplementary file 1 [file jcm-15-03588-s001.zip › jcm-4241701-supplementary.pdf]

Machine learning models for objective assessment of vascular anastomoses using computational fluid dynamics for surgical skill training

Supplementary material

Supplementary information S1. Detailed description of the methodology of skill training and numerical simulations

The dataset consisted of a random sample of digital anastomosis representations from courses conducted between 2021-2023. Participants included physicians of different experience levels, taking place in accredited training programs and workshops (accreditation: Department of Surgical Research and Techniques, University of Pécs, Hungary). The simulators were case-specific in a way that they were based on 3D scans of actual surgical scenes from different procedures. Simulation cases included arteriovenous fistula operation, eversion carotid endarterectomy on highly bifurcated left carotid artery, and coronary artery bypass graft on the left anterior descending artery (also summarized in Table 1). In some cases, information about the simulator used for creating the anastomosis was lost during processing (not available, marked as N/A in Table 1). Standard instrumentation was provided, but own instruments were allowed to be used. The simulators were equipped with plastic tubes of similar size and consistency. Artery imitations were made of a realistic silicone material with 3-5 mm inner diameter. 3D-printed simulators and vessel imitations were manufactured by ME3D-Graft Ltd., Hungary

Before the course, learners had compulsory practical training on basic surgical techniques such as tissue handling, knot tying, instrument application, etc. Instructions suggested to perform the task according to the surgical standards of that procedure. Samples of an ideal solution were not provided, so that the end result reflected the participant's concept of the best technique and approach to the given simulator/case, and ultimately their ability to perform the simulation.

Vascular end-to-side anastomoses were performed with 6/0 polypropylene running sutures. After completion every second consecutive segment of anastomoses. The scanned point cloud was converted into an unstructured 3D mesh and extended using 25 mm regular tubes at all boundaries (SpaceClaim, Ansys Inc. USA). Self-iterations and holes have been removed from the mesh (Meshmixer, Autodesk Inc, USA; Geomagic Wrap, 3DSystems Inc., USA). Inner diameters were scaled uniformly to 3 mm to create comparable volumes. Volume meshing was performed using tetrahedral meshing with 6 prism layers and at least 300.000 cells (ANSYS 21 R2 software, Ansys Inc, USA).

The CFD simulations were carried out as described by Wlasitsch et al [32]. Physiological pulsatile velocity inlet profiles were used with 100 Hz sampling frequency. To enable the simulation equations to converge, two cardiac cycles were simulated using 0.01 s timestep size with a maximum of 10 iterations per step. To shorten simulation time, constant pressure was assumed at the outflows. Blood was considered to have a fluid density of 1.060 g/cm3, non-

Newtonian viscosity was modeled obeying the Carreau model (Supplementary material Eq. 1). [33] Blood flow was modeled with laminar flow in 3D, governing equations were the continuity and Navier-Stokes equations (Supplementary material Eq. 2-3), solver: ANSYS Fluent 21 R2, Ansys Inc, USA.

Table S1. Hyperparameter search space.

Algorithm	Parameter	Search space
**(Scaling)**		Quantile transformation<br>Min-Max scaling<br>Yeo-Johnson transformation
**Ridge**	alpha	$logU(10^{-5}, 10^{2})$
**PLS**	n_components	$\{1..15\}$
**SVR**	kernel	{linear;2ndpolynomial;radial}
	c	$\log U(10^{-5}, 10^{5})$
	epsilon	$\log U(10^{-5}, 10^{5})$
**RF**<br>**XT**	n_estimators	200
	max_depth	$\{1..15\}$
	max_features	$U(0,1)$
	min_samples_split	$\{5..100\}$
**XGBoost**	n_estimators	200
	learning_rate	$U(10^{-4}, 1)$
	max_depth	$\{1..15\}$
	subsample	$\{0.5,0.6\}$

Abbreviations and symbols. PLS: Partial Least Squares; SVR: Support Vector Regression; RF: Random Forest; XT: eXtremely randomized Trees; XGBoost: eXtreme Gradient Boosting; U: uniform distribution; logU: log-uniform distribution

Table S2. Spearman's correlations among different CFD and trainee characteristics.

		Pilot		Main study	
**Var1**	**Var2**	**R_s**	**p**	**R_s**	**p**
	Helicity$_{max}$	-0.61	<0.001	-0.43	<0.001
	v_{max}	**-0.57**	**0.001**	**-0.40**	**<0.001**
	E_{loss}	-0.56	0.001	-0.36	<0.001
	p_{max}	**-0.55**	**0.002**	**-0.46**	**<0.001**
	dp$_{in2-out}$	-0.53	0.003	-0.42	<0.001
	Vorticity$_{max}$	**-0.52**	**0.003**	**-0.37**	**<0.001**
	Strain$_{max}$	-0.52	0.003	-0.38	<0.001
	v_{avg}	**-0.50**	**0.005**	**-0.25**	**0.019**
	TAWSS	-0.45	0.014	-0.39	<0.001
**Years since graduation**	dp$_{in1-out}$	**-0.44**	**0.014**	**-0.28**	**0.010**
**("Experiencee")**	WSSG$_{mag}$	-0.43	0.018	-0.30	0.005
	WSS$_{trans}$	**-0.40**	**0.028**	**-0.36**	**<0.001**
	**Rank**	0.37	0.044	0.20	0.063
	RRT	**0.49**	**0.006**	**0.37**	**<0.001**
	Reynolds$_{max}$	-0.13	0.503	0.16	0.14
	v_{out}	-0.14	0.446	**-0.10**	0.367
	Q_{in2}	0.17	0.366	0.10	0.394
	Q_{in1}	0.22	0.232	0.23	**0.033**
	E_{eff}	0.32	0.087	0.20	0.071
	OSI	-0.13	0.522	-0.08	0.494
	WSS$_{trans}$	-0.82	<0.001	-0.79	<0.001
	v_{avg}	**-0.79**	**<0.001**	**-0.66**	**<0.001**
	WSS	-0.72	<0.001	-0.75	<0.001
	WSSG$_{mag}$	**-0.71**	**<0.001**	**-0.76**	**<0.001**
	Helicity$_{max}$	-0.70	<0.001	-0.67	<0.001
	Vorticity$_{max}$	**-0.70**	**<0.001**	**-0.71**	**<0.001**
	Strain$_{max}$	-0.70	<0.001	-0.70	<0.001
	dp$_{(in2-out)}$	**-0.67**	**<0.001**	**-0.74**	**<0.001**
	dp$_{(in1-out)}$	-0.61	<0.001	-0.70	<0.001
**Mean rank of sutures per trainee**	p_{max}	**-0.60**	**<0.001**	**-0.69**	**<0.001**
**("Rank")**	E_{loss}	-0.59	<0.001	-0.69	<0.001
	v_{max}	**-0.52**	**0.003**	**-0.62**	**<0.001**
	**Experience**	0.37	0.044	0.20	0.063
	Q_{in2}	**0.39**	**0.034**	**0.31**	**0.004**
	Q_{in1}	0.45	0.012	0.42	<0.001
	E_{eff}	**0.49**	**0.006**	**0.51**	**<0.001**
	RRT	0.73	<0.001	0.73	<0.001
	OSI	0.03	0.873	-0.14	0.204
	Reynolds$_{max}$	-0.06	0.769	-0.12	0.278
	v_{out}	-0.07	0.730	-0.09	0.41

Table S3. Descriptive statistics of numeric variables on the full dataset.

	Unit	Min	Mean	Median	Max	IQR	Range	SD	Skewness
**Experience**	[year]	0.00	4.75	3.00	22.00	4.00	22.00	4.49	1.97
**Rank**	-	3.29	5.53	5.49	8.12	1.56	4.83	1.14	0.06
**OSI**	-	0.00	0.02	0.01	0.04	0.01	0.03	0.01	1.02
**RRT**	[1/Pa]	0.43	0.76	0.72	1.49	0.35	1.06	0.22	0.80
**TAWSS**	[Pa]	0.83	1.58	1.59	2.57	0.75	1.73	0.44	0.21
$\mathbf{WSS_{trans}}$	[Pa]	0.08	0.24	0.24	0.40	0.11	0.32	0.08	0.20
**WSSG**	[Pa]	270.84	742.78	718.60	1381.13	408.96	1110.29	288.73	0.50
$\mathbf{Strain_{max}}$	-	791.15	1940.29	1763.86	3925.87	1214.07	3134.73	798.03	0.56
$\mathbf{Helicity_{max}}$	[m/s2]	3.89	27.11	20.68	74.43	27.71	70.54	18.65	0.83
$\mathbf{Vorticity_{max}}$	[1/s]	772.35	2018.66	1880.01	4223.97	1314.38	3451.62	851.82	0.58
$\mathbf{Reynolds_{max}}$	-	9.49	10.70	10.61	12.53	0.69	3.05	0.61	0.82
$\mathbf{E_{loss}}$	[Pa]	0.01	0.03	0.03	0.05	0.01	0.03	0.01	0.32
$\mathbf{E_{eff}}$	-	0.21	0.92	0.96	0.98	0.01	0.77	0.14	-3.94
$\mathbf{p_{max}}$	[Pa]	63.20	7454.84	7856.47	7905.74	23.40	7842.54	1480.03	-3.92
$\mathbf{v_{avg}}$	[mm/s]	58.06	65.80	66.19	72.44	3.19	14.38	2.66	-0.385
$\mathbf{v_{max}}$	[mm/s]	179.70	209.58	200.40	277.46	40.66	97.76	25.63	0.876
$\mathbf{v_{out}}$	[mm/s]	94.95	101.61	101.27	111.58	0.32	16.63	2.04	1.682
$\mathbf{Q_{in1}}$	[mg/s]	0.32	0.34	0.33	0.44	0.01	0.12	0.02	0.00
$\mathbf{Q_{in2}}$	[mg/s]	0.33	0.38	0.38	0.49	0.00	0.16	0.03	0.00
$\mathbf{dp_{in2-out}}$	[Pa]	53.78	78.48	76.98	125.26	22.19	71.48	14.75	0.49
$\mathbf{dp_{in1-out}}$	[Pa]	52.41	68.03	67.83	93.71	10.35	41.30	8.41	0.75

Equations

$$\mu = \mu_\infty + (\mu_0 - \mu_\infty)[1 + (\lambda\dot{\gamma})^2]^{\frac{n-1}{2}} \quad \text{(Eq. 1)}$$

$$\nabla \cdot \vec{u} = 0 \quad \text{(Eq. 2)}$$

$$\frac{\partial \vec{u}}{\partial t} + (\vec{u} \cdot \nabla)\vec{u} = -\frac{\nabla P}{\rho} + \nu\nabla^2\vec{u} \quad \text{(Eq. 3)}$$

$$TAWSS = \frac{1}{t}\int_0^T |\overrightarrow{\tau_w}| dt \quad \text{(Eq. 4)}$$

$$OSI = \frac{1}{2}\left(1 - \frac{\int_0^T |\overrightarrow{\tau_w} dt|}{\int_0^T |\overrightarrow{\tau_w}| dt}\right) \quad \text{(Eq. 5)}$$

$$RRT = \frac{1}{(1-2\times OSI)\times WSS} \quad \text{(Eq. 6)}$$

$$WSS_{\text{trans}} = \frac{1}{T}\int_0^T \left|\overrightarrow{\tau_w}\left(\frac{\vec{n}\times\int_0^T \overrightarrow{\tau_w} dt}{\left|\int_0^T \overrightarrow{\tau_w} dt\right|}\right)\right| dt \quad \text{(Eq. 7)}$$

$$E_{\text{loss}} = \sum_{\text{inlet}}(P_i \cdot Q_i) - \sum_{\text{outlet}}(P_o \cdot Q_o) \quad \text{(Eq. 8)}$$

$$E_{\text{loss}} = \sum_{\text{inlet}} Q_i \cdot \Delta P \ ; \ (\because \sum_{\text{inlet}} Q_i = \sum_{\text{outlet}} Q_o) \quad \text{(Eq. 9)}$$

$$E_{\text{eff}} = \frac{\sum_{\text{outlet}}(P_o \cdot Q_o)}{\sum_{\text{inlet}}(P_i \cdot Q_i)} \quad \text{(Eq. 10)}$$

$$p(t;\alpha) = (p_0 - p_f)e^{-\alpha t} + p_f \quad \text{(Eq. 11)}$$

Where μ is the blood's dynamics viscosity, γ is the wall shear rate, $\lambda = 3.313s$ and $n = 0.3568$ are constants. $\vec{u}$ denotes the fluid velocity vector, ρ is mass density, ν is kinematic viscosity, t is time. $\overrightarrow{\tau_w}$ is the instantaneous WSS vector, $\vec{n}$ is the surface normal, P is pressure, Q is flow rate. p_0, p_f, t are the initial probability, end probability, generation, and $\alpha = 0.4$ is the adaptive rate.

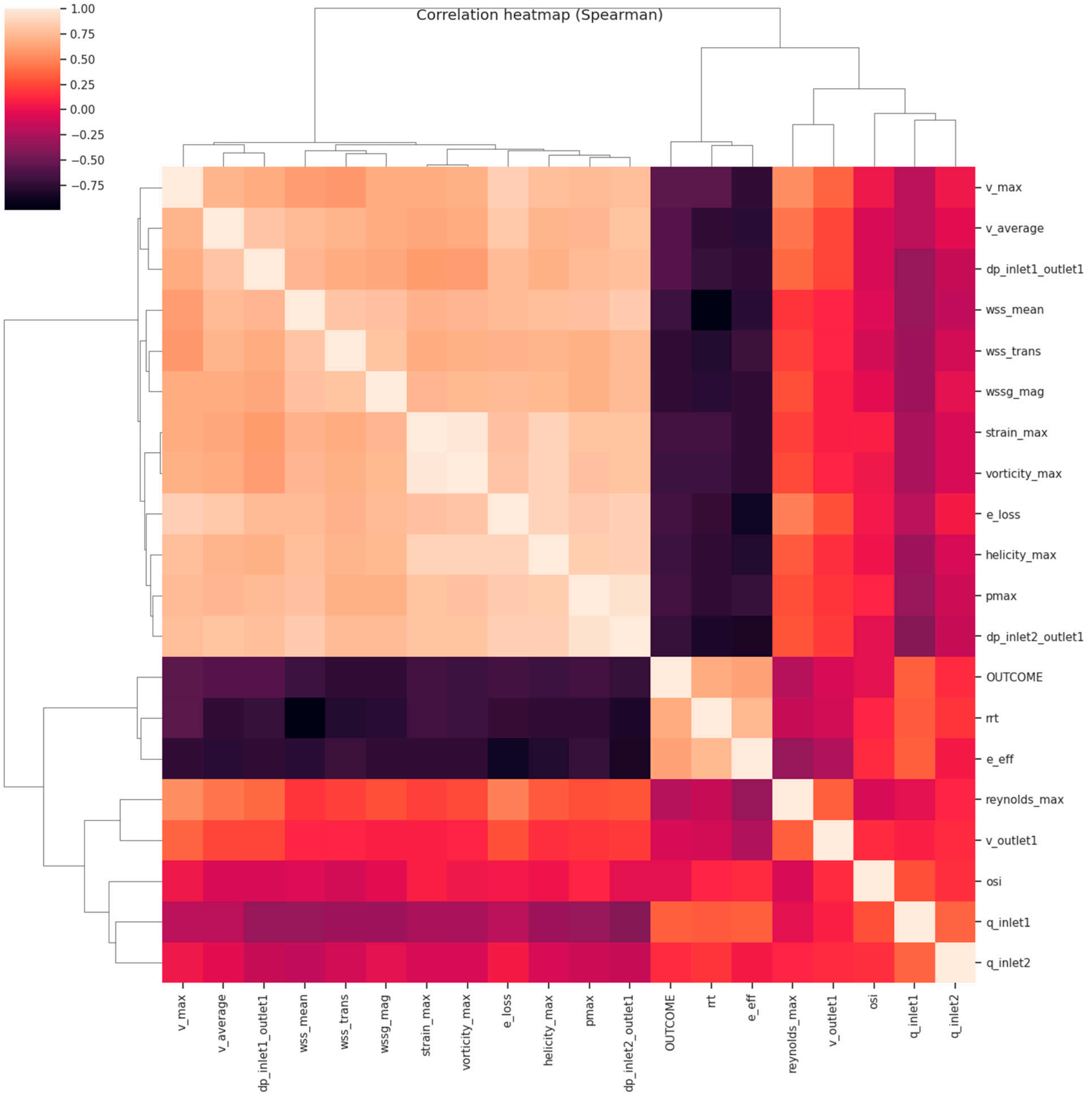

Figure S1. Spearman's correlation heatmap of the model variables.
